# Autophagic Markers in Chordomas: Immunohistochemical Analysis and Comparison with the Immune Microenvironment of Chordoma Tissues

**DOI:** 10.3390/cancers13092169

**Published:** 2021-04-30

**Authors:** Georgia Karpathiou, Maroa Dridi, Lila Krebs-Drouot, François Vassal, Emmanuel Jouanneau, Timothée Jacquesson, Cédric Barrey, Jean Michel Prades, Jean Marc Dumollard, David Meyronet, Jean Boutonnat, Michel Péoc’h

**Affiliations:** 1 Pathology Department, University Hospital of Saint-Etienne, 42055 Saint-Etienne, France; maroa.dridi@etu.univ-st-etienne.fr (M.D.); j.marc.dumollard@chu-st-etienne.fr (J.M.D.); michel.peoch@chu-st-etienne.fr (M.P.); 2 Pathology Department, University Hospital of Grenoble, 38700 Grenoble, France; lkrebsdrouot@chu-grenoble.fr (L.K.-D.); jboutonnat@chu-grenoble.fr (J.B.); 3 Neurosurgery Department, University Hospital of Saint-Etienne, 42055 Saint-Etienne, France; francois.vassal@chu-st-etienne.fr; 4 Department of Neurosurgery B, Neurological Hospital Pierre Wertheimer, 69500 Lyon, France; emmanuel.jouanneau@chu-lyon.fr (E.J.); timothee.jacquesson@neurochirurgie.fr (T.J.); 5 Inserm U1052, CNRS UMR5286, «Signaling, Metabolism and Tumor Progression» The Cancer Research Center of Lyon, 69373 Lyon, France; 6 Claude Bernard University, Lyon 1, 69100 Lyon, France; c.barrey@wanadoo.fr (C.B.); david.meyronet@chu-lyon.fr (D.M.); 7 Department of Anatomy, Faculté de Médecine Lyon-Est, Université de Lyon, Université Claude Bernard Lyon 1, 69100 Lyon, France; 8 Department of Spine and Spinal Cord Surgery, Neurological Hospital Pierre Wertheimer, 69500 Lyon, France; 9 Head and Neck Surgery Department, University Hospital of Saint-Etienne, 42055 Saint-Etienne, France; jean.michel.prades@univ-st-etienne.fr; 10 East Pathology Institute, Hospices Civils de Lyon, 69677 Lyon, France; 11 Cancer Research Center of Lyon, Cancer Cell Plasticity Department, 69373 Lyon, France

**Keywords:** LC3B, ATG16L1, p62, M6PR, PD-L1, CD8, notochord

## Abstract

**Simple Summary:**

In contrast to normal notochords, autophagic factors are often present in chordomas. Furthermore, PD-L1+ immune cells also express LC3B, suggesting the need for further investigations between autophagy and the immune microenvironment.

**Abstract:**

Chordomas are notably resistant to chemotherapy. One of the cytoprotective mechanisms implicated in chemoresistance is autophagy. There are indirect data that autophagy could be implicated in chordomas, but its presence has not been studied in chordoma tissues. Sixty-one (61) chordomas were immunohistochemically studied for autophagic markers and their expression was compared with the expression in notochords, clinicopathological data, as well as the tumor immune microenvironment. All chordomas strongly and diffusely expressed cytoplasmic p62 (sequestosome 1, SQSTM1/p62), whereas 16 (26.2%) tumors also showed nuclear p62 expression. LC3B (Microtubule-associated protein 1A/1B-light chain 3B) tumor cell expression was found in 44 (72.1%) tumors. Autophagy-related 16‑like 1 (ATG16L1) was also expressed by most tumors. All tumors expressed mannose-6-phosphate/insulin-like growth factor 2 receptor (M6PR/IGF2R). LC3B tumor cell expression was negatively associated with tumor size, while no other parameters, such as age, sex, localization, or survival, were associated with the immunohistochemical factors studied. LC3B immune cell expression showed a significant positive association with programmed death-ligand 1 (PD-L1)+ immune cells and with a higher vascular density. ATG16L1 expression was also positively associated with higher vascular density. Notochords (*n* = 5) showed different immunostaining with a very weak LC3B and M6PR expression, and no p62 expression. In contrast to normal notochords, autophagic factors such as LC3B and ATG16L1 are often present in chordomas, associated with a strong and diffuse expression of p62, suggesting a blocked autophagic flow. Furthermore, PD-L1+ immune cells also express LC3B, suggesting the need for further investigations between autophagy and the immune microenvironment.

## 1. Introduction

Chordomas are rare bone tumors, accounting for 1.4% of primary bone malignancies and showing a median overall survival of 7 years; they are assumed to derive from notochordal remnants probably driven by brachyury activation [1]. These malignant tumors, primarily treated with surgery and/or radiotherapy, are notably resistant to chemotherapy [2]. The reason for their chemoresistance is unknown. One mechanism that tumor cells use to survive during adverse conditions is autophagy [3], the discovery of which led to a 2016 Nobel Prize award for Yoshinori Ohsumi [4]. It is a process characterized by the formation of vesicles, autophagosomes, engulfing cellular constituents and leading them to degradation and recycling by fusion with the lysosomes [3]. It is one of the first responses in tumor cells exposed to chemotherapy, as it removes damaged proteins and organelles and generates energy [5]. Thus, autophagy often acts as a cytoprotective mechanism, and therefore chemotherapeutic drugs are used in combination with autophagy inhibitors in clinical trials [5]. Furthermore, lysosomes, that receive extracellular/cell surface molecules by endocytosis and intracellular components by autophagy, are important in drug resistance as they isolate chemotherapeutic drugs [6]. The principal morphologic feature of chordomas, already described by Virchow, is their cytoplasmic vacuoles, accounting for their bubbled cytoplasm and explaining the description Virchow gave to chordoma cells: “physaliphorous” (from the Greek words physalis = bubble and phorous = bearing) [2]. The exact nature of these vacuoles remains unknown, but they are considered lysosome-related organelles [7]. Furthermore, brachyury, the main gene implicated in chordomas pathology [2], has been shown to induce autophagy in glioblastoma cell lines [8]. Despite this indirect evidence of chordomas association with autophagy, to the best of our knowledge, the presence of this mechanism has never been studied in chordoma tissues. 

Furthermore, the immune microenvironment is important for all tumors, even for sarcomas, with recent studies suggesting prognostic significance of immune cells, notably B cells, in soft tissue sarcomas [9]. Still, controversial findings as to the role of the immune microenvironment of chordomas [10,11,12], despite immunotherapy, could be considered a possible option for chordoma patients [13]. Moreover, there is recent evidence suggesting that the autophagic machinery of the tumor-associated lymphocytes, controls their own phenotype [14], implying an association of the autophagy with the immune microenvironment as well. This association has not been previously studied in chordomas.

Thus, the aim of this study is to investigate the possible presence of autophagic markers in a large series of chordomas and to correlate them with the immune microenvironment of these tumors.

## 2. Materials and Methods

This is a multicenter retrospective study of 61 patients diagnosed with chordoma of the conventional subtype, between 2000 and 2020, based on clinicoradiological data, typical morphological features and S100/cytokeratins expression, and confirmed in reassessment by a specialized soft-tissue pathologist (MP) and by brachyury expression. The local ethics committee approved the study (IRBN702020/CHUSTE). Tumor localization and size, treatment type, tumor recurrence and overall and progression-free survival were retrieved from medical records. 

**Figure 1:**
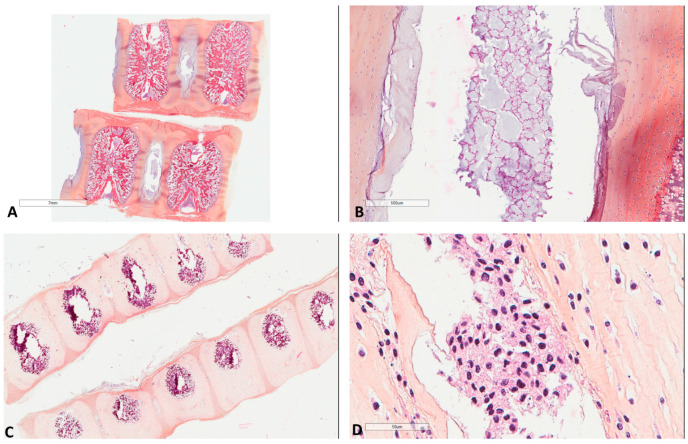
Morphology of fetal notochords. (**A**) Low magnification highlighting their intervertebral location (39 weeks of gestation, Hematoxylin, Eosin, Safran (HES) ×3). (**B**) Intermediate magnification showing their typical morphological resemblance to chordomas (39 weeks of gestation, HES ×40). (**C**) Low magnification highlighting their intervertebral location of another case (17 weeks of gestation, HES ×8). (**D**) High magnification showing their typical morphological resemblance to chordomas (17 weeks of gestation, HES ×400).

Immunohistochemistry was performed in formalin-fixed paraffine-embedded 4-μm thick full tumor sections using an automated staining system (OMNIS, Dako-Agilent, Santa Clara, CA, USA). Primary antibodies used were: LC3B (Rabbit monoclonal, ab192890, abcam, dilution 1/1000, pH 6, 20 min), SQSTM1 (sequestosome1)/p62 (Rabbit monoclonal, ab109012, abcam, dilution 1/2000, pH 6, 20 min), ATG16L1 (Rabbit monoclonal, ab195242, abcam, dilution 1/1000, pH 9, 20 min) and M6PR (cation independent) (Rabbit monoclonal, ab124767, abcam, dilution 1/2000, pH 6, 20 min). Positive immunoreactions were visualized using 3,3’-diaminobenzidine as the chromogenic substrate. The antibodies had been initially tested in a large variety of normal and neoplastic tissues to decide the best immunohistochemical protocol, giving no background staining and a range of staining intensities. Thereafter, nerve fiber and normal tonsillar tissue were used as positive controls for LC3B and p62/M6PR, respectively, while omission of the primary antibody was used as negative control. Given the histogenetic association of chordomas to notochord, 5 normal notochords (Figure 1) were also immunohistochemically studied for LC3B, p62 and M6PR, for comparison with the presumed tissue of origin of chordomas.

LC3B and M6PR staining was presented as cytoplasmic punctae and according to the density of dots per cell. This was recorded as negative (intensity score 0, no staining or ≤10 dots per cell); mild (intensity score 1, 11–20 dots per cell); moderate (intensity score 2, >20 dots per cell without clusters), and strong (intensity score 3, >20 dots per cell with clusters) [15]. The intensity of p62 and ATG16L1 staining was recorded as negative (intensity score 0), weak (intensity score 1), moderate (intensity score 2), and strong (intensity score 3). The percentage of positive cells was recorded from 0 to 100% and presented as the H score (percentage of positive cells × intensity). P62 can also show nuclear expression and tumors with at least 5% p62 nuclear staining were considered positive for nuclear expression, as previously suggested [16].

The tumors were also studied for the immunohistochemical expression of (work under submission): PD-L1 (22C3, Dako Agilent, 1/40), CD8 (C8/144B, Dako Agilent, 1/100), CD20 (L26, Dako Agilent, 1/200), CD163 (10D6, Novocastra, 1/200), CD34 (QBEnd10, Dako Agilent, 1/800) and MECA-79 (MECA-79, Santa Cruz Biotechnology, 1/750). MECA-79 is a factor detecting high endothelial venules; vessels specialized in the transport of lymphocytes [9]. We evaluated the immune cells in a semiquantitative manner (0: no cells, 1: few cells (<10%), 2: moderate number of positive cells (≥10% and <40%), and 3: abundant cells (≥40%). This resulted in low (scores 0 and 1) and high (scores 2 and 3) groups for CD8, CD20 and CD163; and present (score 0) or absent (score 1–3) for PD-L1+ immune cells [17,18]. Quantification of the number of CD34+ and MECA-79+ blood vessels (vascular density) was performed on 5 high power 20× (1 mm^2^) fields per section, and these were counted and averaged, as previously proposed [19] while their median value was used as a cut-off for the classification into two groups. 

Data were analyzed using StatView software (Abacus Concepts, Berkley, CA, USA). We used the χ^2^ test to explore any relationship between two groups for categorical data, and factorial analysis of variances (ANOVAs) to consider the effect of at least one factor on a continuous parameter studied. Simple regression analysis was used to explore a possible relationship between two continuous parameters. Survival probability was estimated by Kaplan–Meier analysis with log-rank product limit estimation. For all analyses, statistical significance was indicated at a *p* value of <0.05.

## 3. Results

The cohort (*n* = 61), which is part of our previous study (work under submission), included 37 (60.7%) male and 24 female (39.3%) patients with a mean age at diagnosis of 56.5 (±16.8) and a median of 61 years. Tumors were more often skull chordomas (*n* = 23, 37.7%), followed by sacral (*n* = 21, 34.4%) and mobile spine (*n* = 17, 27.9%) tumors. Patients had been treated with surgery in most cases (*n* = 59, 96.7%), followed by adjuvant therapy in almost half of the cases. Follow up ranged from 2 to 264 months (median 64, mean 92.3±71.7). Recurrences were noted in 46 patients (75.4%). Thirteen patients (*n* = 13, 21.3%) died of disease. The 5-year and 10-year overall survival (log-rank) was 80% and 70% respectively. 

**Figure 2:**
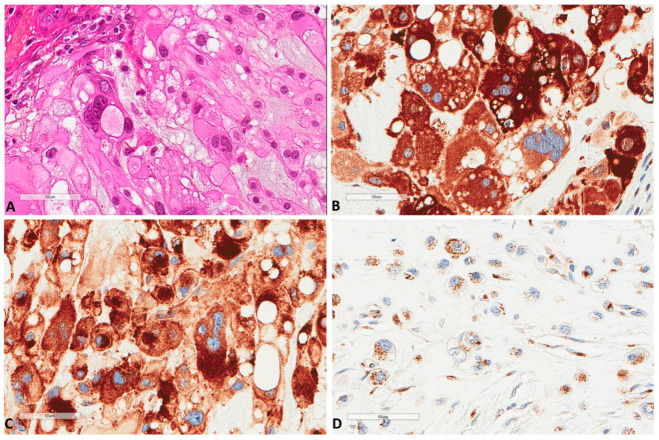
P62 and M6PR expression in chordomas. (**A**) The morphology of a chordoma tissue (×400). (**B**) P62 strong express Scheme 400. (**C**) Same focus for M6PR expression (×400). (**D**) Another chordoma with lower M6PR expression (×400).

The immunohistochemical study (Table 1 and Table S1) showed that all chordomas strongly expressed (Figure 2) cytoplasmic p62 (*n* = 61, median H score 300); thus, no further statistical correlations were performed for this factor. 

**Figure 3:**
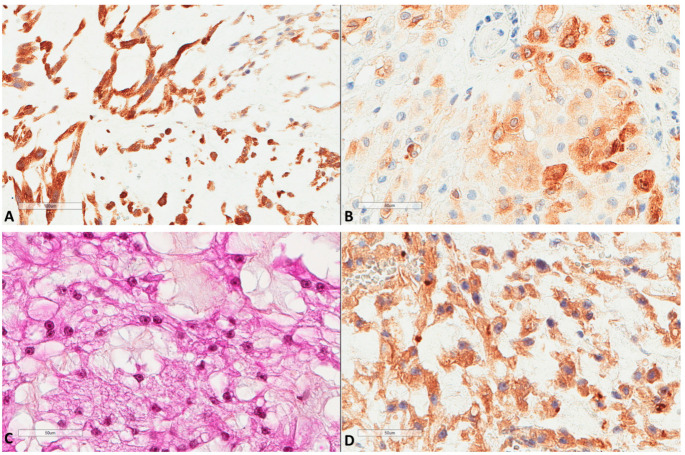
LC3B and ATG16L1 expression in chordomas. (**A**) LC3B strong expression of a chordoma (×200). (**B**) LC3B mild expression of another case (×400). (**C**) Another chordoma tissue (HES ×400). (**D**) ATG16L1 expression of the latter chordoma (×400).

Sixteen (*n* = 16, 26.2%) tumors also showed nuclear p62 expression. Similarly, all tumors (*n* = 61) expressed M6PR (Figure 2), and expression was homogenous in each tumor, thus, three groups of intensity score 1 (*n* = 23, 37.7%), 2 (*n* = 21, 34.4%), or 3 (*n* = 17, 27.9%) were used for further analyses. LC3B tumor cell expression (*n* = 61) was found in 44 (72.1%) tumors (Figure 3). 

**Figure 4:**
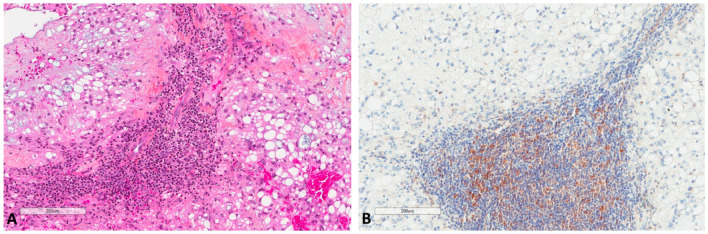
LC3B expression in immune cells of chordomas. (**A**) Immune cells in chordoma stromal tissue (HES ×100). (**B**) LC3B expression in immune cells at the same focus (×100).

H score ranged from 0 to 100, with a median of 10 and a mean of 16.2 (±22.5). The median H score was used as a cut-off value to classify tumors into low (≤10) or high (>10) expression. LC3B expression by immune cells (*n* = 61) inside tumor stroma (Figure 4) was found in 18 (29.5%) tumors, with the H score ranging from 0 to 50 (median 0 and mean 10.7 ± 23.4).

ATG16L1 (*n* = 55, due to technical issues) was also expressed (Figure 3) by most tumors (*n* = 42, 76.4%) with a median H score of 100 (0–300) and a mean of 106.7 ± 85.2. The median cut-off was used to classify tumors into low (≤100) or high (>100) expression. 

We found that LC3B tumor cell expression (χ^2^ test) was negatively associated with tumor size (*p* = 0.03, χ^2^ = 4.5), where tumor size was available for 29 tumors and the median tumor size (43 mm) was used as the cut-off value for the χ^2^ test. Expression was marginally associated with localization, since it was less often found in sacral tumors (*p* = 0.07, χ^2^ = 5.2), but this probably reflected tumor size, because sacral tumors were larger than in other localizations (*p* = 0.02, χ^2^ = 7). None of the other parameters, such as age, sex, localization, or size were associated with the immunohistochemical factors studied.

**Figure 5:**
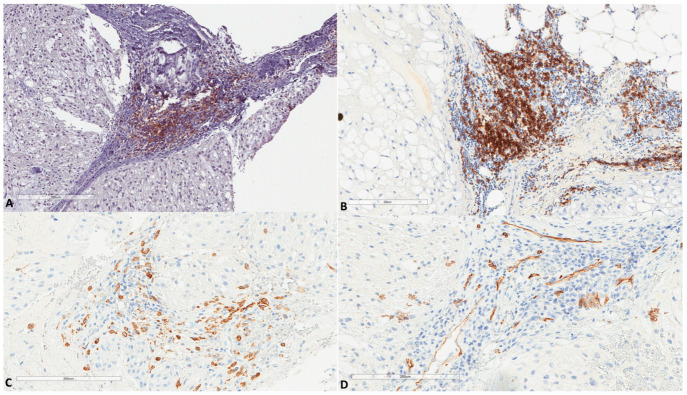
(**A**) PD-L1 expression in immune cells of chordomas (HES ×100, same focus as in Figure 4). (**B**) Another chordoma with high infiltration by B cells (×100, CD20+). (**C**) Infiltration by CD163+ macrophages (×200, same focus as in Figure 4). (**D**) Vascular channels as assessed by CD34 (×200, same focus as in Figure 4).

Autophagic factors studied herein were compared (Figure 5 and Table 2) with our previous data regarding the immune micro-environment of chordomas (work under submission). The scheme of low/high expression, as mentioned above for each factor, was used for statistical analyses by χ^2^ test. LC3B immune cell expression showed a marginal and negative association with CD20 B cells presence (*p* = 0.07, χ^2^ = 3). It showed a significant positive association with PD-L1+ immune cells (*p* = 0.001, χ^2^ = 9.7). It also showed a strong positive correlation with high vascular density as studied by CD34 (*p* = 0.0004, χ^2^ = 12.4). ATG16L1 expression was also positively associated with vascular density (*p* = 0.01, χ^2^ = 6.4), while no other association was found for this marker. The presence of CD163 positive macrophages was not associated with the present factors. The presence of high endothelial venules, assessed by the MECA-79 antibody, was not associated with any of the factors studied; however, it showed a trend (*p* = 0.06, χ^2^ = 3.4) for a negative association with p62 nuclear expression. A strong trend (*p* = 0.05, χ^2^ = 3.7) between p62 nuclear expression and the presence of lesser B cells was also noted.

The immunohistochemical factors currently studied were not associated with each other (χ^2^ test); however, a strong trend (*p* = 0.05, χ^2^ = 5.7) was found between M6PR and p62 nuclear expression: tumors with p62 nuclear expression showed milder M6PR expression. 

Survival analysis showed no prognostic significance for the autophagic immunohistochemical factors studied.

**Figure 6:**
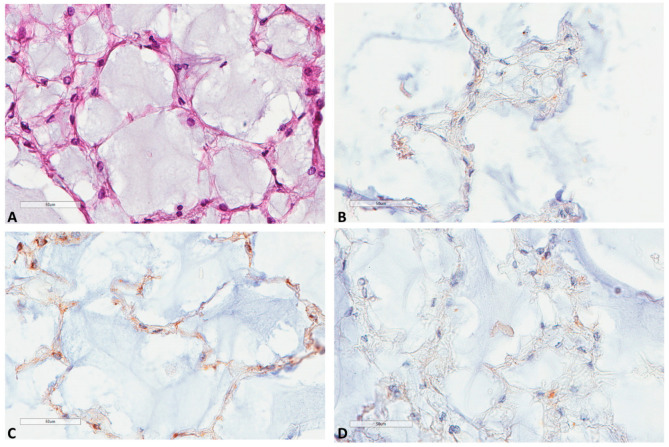
LC3B, M6PR and p62 expression in notochords. (**A**) Notochord morphology (39 weeks of gestation, HES ×400). (**B**) LC3B very mild expression at the same focus (×400). (**C**) M6PR mild expression at the same focus (×400). (**D**) p62 negative staining at the same focus (×400).

Regarding notochords immunostaining (Figure 6), five notochords from fetal autopsies (ages of 14, 17, 23, 23 and 39 weeks of gestation) showed the same pattern in all cases: a very weak LC3B and M6PR expression, and no p62 expression.

## 4. Discussion

This is the first study, to the best of our knowledge, examining the current immunohistochemical factors in chordoma tumor tissues. We found that all chordomas strongly and diffusely express p62, a receptor of autophagic cargos. This factor binds and transports the targets to the autophagosome by interacting with LC3, and then p62 itself is degraded during autophagolysosome cargo degradation; thus, reduced levels of p62 are typically used as a surrogate marker of an activated autophagy pathway [20]. Therefore, the constant and high expression of p62 in chordomas found herein probably reflect a blocked autophagic degradation. Simultaneously, we found that most tumors showed LC3B expression, which is used as a surrogate marker of autophagic vesicles. This combination of LC3B and p62 expression is suggested to represent activated but blocked, downstream autophagic machinery [15]. Similarly, the autophagy-related 16‑like 1 (ATG16L1) protein was expressed in most tumors, further supporting the presence of autophagic factors in chordoma tissues. ATG16L1 is one of the critical initial steps of the autophagic activation, since it is the mediator that specifies the site of LC3 lipidation [21]. Additionally, it provides a link between autophagy and immune regulation, since it interacts with the cytokine receptor’s intracellular domain [21]. Thus, our results are in favor of a probably activated but blocked autophagic mechanism in chordomas, resulting in accumulated autolysosomes in tumor cells cytoplasm. This could probably explain the chemoresistance of these cells. In line with this assumption, enhanced autophagy has been shown to protect osteosarcoma cells against chemotherapeutic stress [22,23]. Our results are also in line with previous studies in cell lines, where it was shown that lysosomal vacuoles in chordomas do not harbor acidic pH 7, and thus their function is impaired, which could explain the accumulation of p62. Furthermore, we show that staining of tumor tissues is different from staining seen in notochords, further supporting a dysregulated mechanism in the tumoral setting. The exact mechanism of activation of autophagy in chordomas is unknown, but a possible association with brachyury activation, which is the main pathogenetic molecular abnormality in chordomas, could be hypothesized since it induces autophagy in gliomas [8]. 

Mannose-6-phosphate receptor (M6PR), also called insulin-like growth factor-2 (IGF-2) receptor is a receptor that leads cell surface/cytoplasmic constituents to endosomal vesicles and to autophagosomes for degradation. The IGF-2R/M6PR, which is considered to act as a tumor growth suppressor, has two forms: a membrane-associated and a soluble one, both of which interact with several ligands, including IGF-2, TGFβ and lysosomal enzymes [24]. It was found in all chordomas, showing strong expression in almost one third of them. Another receptor of the same family, the IGF1R was also found to be expressed by most chordomas [25]. It has been shown that M6PR acts as a link between autophagy, chemotherapy and immunotherapy, since autophagy controls its traffic between the cytoplasm and the cell surface, where it augments T cell cytotoxic activity against tumor cells [26,27,28]. Furthermore, after chemotherapy, IGF2 confers resistance correlated with enhanced autophagy when expressed at elevated levels in osteosarcomas [23]. We did not find any association between M6PR and the immune microenvironment of chordomas, which could be explained by the cytoplasmic accumulation of this marker inside tumor cells. Interestingly, its expression was milder for tumors with p62 nuclear expression. 

P62 harbors nuclear import/export signals, but the role of nuclear p62 is unknown [29]. Recent evidence suggests that p62 nuclear retention is favored by the inhibition of exportin 1, and that its nuclear retention enhances the expression of innate immune response related genes [29]. It has been also shown in virus-transformed cells that inhibition of autophagy leads to p62 nuclear accumulation, which in turn leads to reactive oxygen species (ROS)-induced DNA damage and proteasomal degradation of DNA repair proteins [30]. In previous immunohistochemical studies of p62 expression, nuclear expression has been also observed; in endometrial cancer, almost half of the cases showed nuclear expression, and with high cytoplasmic expression associated with absent p62 nuclear expression, denoted an adverse prognosis [16]. Similarly, lower p62 nuclear is associated with poorer survival in oral cancer patients [15]. In lung cancer, when both cytoplasmic and nuclear p62 expression were found, this signified adverse prognosis [20]. All these findings show that p62 nuclear expression is not a fortuitous event, rather, there is a pathophysiological importance for its nuclear accumulation that warrants further investigation. In the current study, its nuclear presence was marginally associated with lesser B cells and lesser high endothelial venules, suggesting a role in regulating this part of the immune microenvironment.

Another finding of our study is that some of the tumors harbored immune cells strongly expressing LC3B and that these cells were PD-L1+ immune cells. To the best of our knowledge, the role of autophagy inside the immune microenvironment of any tumor has not been yet elucidated. However, recent evidence suggests that autophagy is actively implicated in tumor infiltrating lymphocyte activity, and when T cells live inside tumors with elevated extracellular potassium, this reduces the uptake of local nutrients by these lymphocytes, leading to activation of their autophagy [14]. This autophagy activation in T cells, in turn, leads to less factors necessary for epigenetic remodeling, thus leading to a more stem cell-like and less differentiated/effector phenotype of these lymphocytes [14]. Moreover, LC3B+ autophagosomes released by tumor cells in the form of extracellular vesicles, correlate significantly with up-regulation of PD-L1 in matched monocytes from malignant effusions, also suggesting an immunosuppressive mechanism of autophagy in the tumor microenvironment [31]. Thus, our finding of LC3B expression in PD-L1+ immune cells in chordomas probably reflects starvation conditions and autophagic activation in these cellular subpopulations. It is worth noticing that autophagosomes and lysosomes have been found to contain major histocompatibility complex (MHC)-I molecules in pancreatic adenocarcinoma cells, preventing them from being expressed on the cell surface and thus from activating cytotoxic T cells [32,33]. Thus, our data add to the notion of autophagy being implicated in the immune tumor microenvironment and will prompt further investigation.

Our study has limitations associated with its retrospective nature. The main limitation is the investigation of these factors only by immunohistochemical means, where autophagy is a flow, and should also be studied functionally. However, our approach is warranted when a large tissue series of rare diseases, such as chordomas, are needed.

## 5. Conclusions

To conclude, we study for the first time, a large series of chordoma tissues for autophagic markers and compare them with their expression in notochords and with the tumor immune microenvironment. We show that autophagic factors, such as LC3B and ATG16L1, are often present in chordomas, associated with a strong and diffuse expression of p62, suggesting a blocked autophagic flow, in contrast to normal notochords. Furthermore, PD-L1+ immune cells also express LC3B, suggesting the need for further investigations between autophagy and the immune microenvironment.

## Figures and Tables

**Table 1:** Immunohistochemical analysis.

Parameter	Values
**LC3B tumor cell expression H score**	
Range	0–100
Median	10
Mean ± SD	16.2 ± 22.5
**LC3B immune cell expression H score**	
Range	0–100
Median	0
Mean ± SD	10.7 ± 23.4
**P62 cytoplasmic tumor cell expression H score**	
Range	0–300
Median	300
Mean ± SD	231.8 ± 89.4
**P62 nuclear tumor cell expression**	
Yes	16, 26.2%
No	45, 73.8%
**ATG16L1 tumor cell expression H score**	
Range	0–300
Median	100
Mean ± SD	106.7 ± 85.2
**M6PR tumor cell intensity score**	
1 (mild)	23, 37.7%
2 (moderate)	21, 34.4%
3 (strong)	17, 27.9%

**Table 2:** Correlation between the immunohistochemical factors studied.

Variables	LC3B in Tumor Cells			LC3B in Immune Cells			ATG16L1 Expression			M6PR Expression				p62 Nuclear Expression		
	*n* = 61			*n* = 61			*n* = 55			*n* = 61				*n* = 61		
	Low	High	*p*, χ^2^	Low	High	*p*, χ^2^	Low	High	*p*, χ^2^	1	2	3	*p*, χ^2^	No	Yes	*p*, χ^2^
**CD20** (*n* = 60)																
Low	35	18	0.7, 0.08	35	18	0.07, 3	34	14	0.9, 0.001	20	18	15	0.9, 0.09	41	12	0.05, 3.7
High	5	2		7	0		5	2		3	2	2		3	4	
**CD8** (*n* = 61)																
Low	25	11	0.7, 0.1	28	8	0.1, 2.2	26	7	0.1, 2.4	12	14	10	0.6, 0.9	29	7	0.1, 2
High	16	9		15	10		13	9		11	7	7		16	9	
**CD163** (*n* = 61)																
Low	23	12	0.7, 0.08	27	8	0.1, 1.7	25	7	0.1, 1.9	13	14	8	0.4, 1.4	25	10	0.6, 0.2
High	18	8		16	10		14	9		10	7	9		20	6	
**Vascular density** (*n* = 61)																
Low	19	9	0.9, 0.009	26	2	**0.0004, 12.4**	22	3	**0.01, 6.4**	11	11	6	0.5, 1.1	22	6	0.4, 0.6
High	22	11		17	16		17	13		12	10	11		23	10	
**PD-L1+ immune cells** (*n* = 61)																
No	32	12	0.1, 2.1	36	8	**0.001, 9.7**	30	9	0.1, 2.3	15	18	11	0.2, 2.9	33	11	0.7, 0.1
Yes	9	8		7	10		9	7		8	3	6		12	5	
**Tumor size (*n* = 29)**																
**<43 mm**	7	7	**0.03, 4.5**	11	3	0.1, 1.7	7	6	0.6, 0.1	5	6	3	0.1, 3.2	11	3	0.7, 0.1
**≥43 mm**	13	2		9	6		8	5		9	2	4		11	4	

Data presented in Table 2 were statistically calculated using the χ^2^ test. Bold denotes statistical significance.

## Data Availability

Data are available upon reasonable request.

## Supplementary Materials

The following are available online at https://www.mdpi.com/article/10.3390/cancers13092169/s1.
