# Supplementary material for: Autophagic Markers in Chordomas: Immunohistochemical Analysis and Comparison with the Immune Microenvironment of Chordoma Tissues
Source: Cancers (Basel). 2021 Apr 30;13(9):2169. doi: 10.3390/cancers13092169 (PMC8124629; doi:10.3390/cancers13092169)
Supplement: Supplementary file 1 [file cancers-13-02169-s001.zip › cancers-1173831-supplementary.pdf]

Table S1. Immunohistochemical results presented as groups for the markers studied.

Immunohistochemical factor	Expression (*n*, %)
**LC3B expression by tumor cells** (*n* = 61)	
Low	41, 67.2%
High	20, 32.8%
**LC3B expression by immune cells** (*n* = 61)	
Low	43, 70.5%
High	18, 29.5%
**ATG16L1 expression** (*n* = 55)	
Low	39, 70.9%
High	16, 29.1%
**M6PR expression** (*n* = 61)	
Mild	23, 37.7%
Moderate	21, 34.4%
Strong	17, 27.9%
**P62 nuclear expression** (*n* = 61)	
No	45, 73.8%
Yes	16, 26.2%
**CD20 immune cells expression** (*n* = 60)	
Low	53, 88.3%
High	7, 11.7%
**CD8 immune cells expression** (*n* = 61)	
Low	36, 59%
High	25, 41%
**CD163 immune cells expression** (*n* = 61)	
Low	35, 57.4%
High	26, 42.6%
**PD-L1 immune cells expression** (*n* = 61)	
No	44, 72.1%
Yes	17, 27.9%
**Vascular density assessed by CD34 expression** (*n* = 61)	
Low	28, 45.9%
High	33, 54.1%
